# Timing is survival: modeling how earlier calls improve cardiac arrest outcomes

**DOI:** 10.3389/fdgth.2025.1695377

**Published:** 2026-01-08

**Authors:** Marius Ole Johansen, Rune Johan Krumsvik, Vegard Slettvoll

**Affiliations:** 1 Faculty of Psychology, Department of Education, University of Bergen, Bergen, Norway; 2 Faculty of Medicine, University of Bergen, Bergen, Norway

**Keywords:** ambulance response time, artificial intelligence, cardiac arrest, survival simulation, wearables

## Abstract

**Introduction:**

Survival after out-of-hospital cardiac arrest decreases by 5%–12% for every minute of delay in treatment. Ambulance response times vary widely across Norway, particularly between urban and rural municipalities. Advances in digital health technologies may encourage earlier patient contact with emergency services, potentially mitigating these delays.

**Methods:**

We analyzed official response time data from four Norwegian municipalities representing diverse geographic contexts (Bergen, Tokke, Lurøy, Sørfold). Using a survival decay function (Equation), we simulated changes in survival probability under scenarios where emergency calls were placed 1, 5, or 10 min earlier than observed.

**Results:**

Baseline survival probabilities varied substantially across municipalities, from 47.7% in Bergen (mean response 10.2 min) to 9.3% in Lurøy (32.8 min). Simulated earlier calls produced marked gains: in Bergen, survival increased from 47.7% to 68.6% with a five-minute advance; in Sørfold, from 19.4% to 27.9%; and in Tokke, from 29.9% to 43.1%. Even modest improvements (1–2 min) yielded meaningful survival benefits.

**Conclusions:**

Geographic disparities in emergency response times strongly influence survival after cardiac arrest. Wearables and AI-based monitoring cannot predict cardiac arrest but may promote earlier recognition of abnormal physiological states and timelier emergency calls. If widely adopted, such technologies could provide substantial survival gains, particularly in rural and remote regions.

## Introduction

Sudden cardiac arrest remains one of the leading causes of death worldwide, claiming hundreds of thousands of lives each year (1–4). In these situations, time is the single most critical determinant of survival. Multiple international studies have consistently demonstrated that each minute without treatment reduces survival probability by an estimated 5%–12% (5–7). Put differently, the likelihood of a patient leaving the hospital alive declines with every passing minute, highlighting the extreme time sensitivity of these events (8). Even small delays in recognizing symptoms or contacting emergency services can be the decisive factor between life and death. While early cardiopulmonary resuscitation (CPR) and defibrillation are well established as life-saving interventions, they are only effective if help is mobilized in time. The first step in the chain of survival, the recognition of cardiac arrest symptoms and immediate contact with emergency services, remains underexplored in quantitative research (9). Yet this initial link is arguably the most fragile; if patients or bystanders fail to act quickly, subsequent interventions cannot compensate for the time lost.

Norway provides a particularly compelling setting for investigating this problem. The country's highly diverse geography encompasses dense urban areas, remote mountain regions, and scattered island municipalities, each with different levels of access to emergency care (10–13). These geographic differences translate into substantial disparities in ambulance response times. In major cities such as Bergen, the mean response time is just over 10 min, whereas in rural or island communities like Sørfold or Lurøy, patients may face delays exceeding 30 min before an ambulance arrives (14). Given that survival rates can decline by up to 10% per minute, such disparities represent not just geographic inconvenience but profound inequities in survival chances across populations.

This raises an urgent question; What if patients could be prompted to contact emergency services even a few minutes earlier? Advances in digital health technologies suggest this may be possible (15–17). Artificial intelligence (AI), wearable sensors, and home monitoring devices cannot predict the exact moment of cardiac arrest (18). However, they are increasingly capable of learning what is “normal” for an individual and detecting concerning deviations such as arrhythmias or ischemic patterns (18–20). In practical terms, these technologies may raise patient *awareness* and *encourage* earlier contact with emergency services by shaving off critical minutes from the time to treatment. Given the steep decline in survival with each passing minute, even marginal gains could translate into substantial improvements in outcomes at the population level.

### Wearables and AI for earlier recognition

Recent advances in digital health technologies offer a promising avenue for reducing delays in contacting emergency services (21–24). Wearable devices such as smartwatches, chest straps, and home-based sensors are increasingly capable of continuous physiological monitoring (16, 25–29). These systems can routinely measure heart rate, detect rhythm irregularities, assess oxygen saturation, and in some cases even provide single-lead electrocardiograms. While such technologies cannot predict the precise onset of sudden cardiac arrest, they can establish individualized baselines and identify deviations that may signal elevated risk (18, 20, 30–32). Prolonged arrhythmias, ischemic-like patterns, or sustained abnormal vital signs may serve as early warnings that justify precautionary action. AI enhances this potential by enabling true personalization (33, 34). Rather than relying on static population-level thresholds, AI systems can learn an individual's typical physiological profile and detect subtle deviations that might otherwise go unnoticed. The value of such alerts is not in deterministic prediction, but in raising awareness among individuals at heightened risk. In practice, this could encourage simple but life-saving adjustments, such as staying closer to a phone, notifying family members, or contacting medical services sooner than they otherwise would have. The potential survival impact of these earlier actions is substantial. As our simulations illustrate, even a single minute gained can yield a measurable increase in survival probability. In municipalities with long baseline ambulance response times, such as remote rural or island regions, the relative benefit of earlier calls is even more pronounced. Digital health technologies cannot erase the challenges imposed by geography, but they may help narrow the window of preventable delay between symptom recognition and the emergency call.

In the present study, we use emergency medical communication center data from four Norwegian municipalities to model how survival probabilities change under different scenarios of earlier emergency calls. Using a survival rate function combined with Monte Carlo simulations, we estimate how survival chances improve if patients were to call for help 1, 5, or 10 min earlier. Our aim is twofold; 1) to quantify the potential survival benefits of earlier calls across diverse geographical settings, and 2) to discuss how emerging digital health technologies could realistically support such time gains.

## Methods

### Data sources

We obtained data on mean ambulance response times from the Norwegian emergency medical communication centers (14). Data were available at the municipality level and represent the average time from the emergency call to ambulance arrival on scene. For this analysis, we selected four municipalities that illustrate contrasting geographic conditions in Norway; Bergen (urban), Tokke (mountainous), Sørfold (northern rural), and Lurøy (island). These municipalities were chosen to represent the diversity of ambulance response challenges in Norway, from highly central to remote and hard-to-reach regions.

### Survival model

Survival probability following acute cardiac arrest was modeled as a function of time to medical intervention. Previous studies have shown that survival decreases by approximately 5%–12% per minute of delay (5–7). Based on this literature, we applied a simple exponential survival function (35), where survival at time *t* is expressed relative to a baseline survival probability $S_{0}$ at time $t_{0}$,

(1) $$S(t)=S_{0}^{m}\times (1-r{)}^{\left(t-t_{0}\right)}$$

where *r* represents the per-minute rate of decline in survival probability, *m* denotes municipality, and $t_{0}$ corresponds to the mean response time reported for each municipality. The exponential decay function was selected because it represents the simplest and most widely used approximation of declining survival with increasing delay. Rather than estimating r from data, we fixed the per-minute decline at 7%, the midpoint of the commonly reported 5%–12% range, to provide a transparent and reproducible baseline for comparing municipalities. This choice ensures that the framework is easily interpretable and modifiable; in practice, researchers or EMS administrators may substitute alternative r values or incorporate additional parameters such as weather conditions, dispatch constraints, or caller characteristics.

### Simulation approach

To evaluate potential survival gains from earlier emergency calls, we simulated scenarios where the emergency call was placed 1, 5, and 10 min before the average response time. This corresponds to shifting *t* backwards relative to $t_{0}$. To account for stochastic variability, we implemented a Monte Carlo simulation procedure (36). The Monte Carlo simulation was not intended to model physiological uncertainty, but to illustrate how simple stochastic variability can be propagated through the survival function. The code is provided as a template that can be extended with empirically informed distributions as more detailed data become available. For each municipality, survival curves were generated by introducing normally distributed noise (μ=0) around the deterministic survival function. This process was then repeated 10,000 times, where we calculated the mean survival probability and the 95% simulation interval (2.5th–97.5th percentiles). For each municipality, we report 1) the baseline survival probability at the mean response time, 2) the estimated survival probability if the call occurred 1, 5, or 10 min earlier, and 3) the simulated survival curves with uncertainty intervals, illustrating the potential gains in survival. All simulations and visualizations were performed in RStudio version 2023.12.1.

## Results

**Figure 1:**
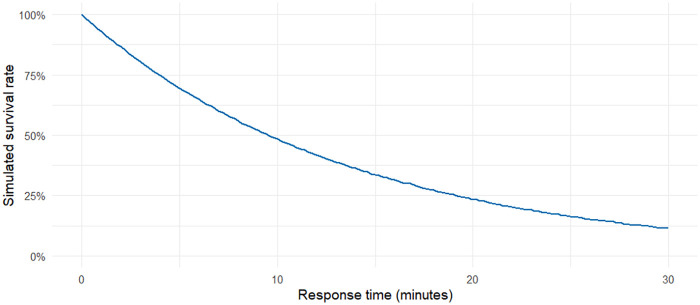
Standard survival rate function for cardiac arrests as a function of response time.

To illustrate the decline in survival probability with increasing ambulance response times, can we implement the standard survival function.

(2) $$S(t)=S_{0}\times (1-r{)}^{\left(t-t_{0}\right)}$$

where $S_{0}$ denotes denotes the baseline survival probability at the observed mean response time $t_{0}$, and *r* represents the estimated per-minute decline in survival. This formulation captures the exponential decay of survival probability with increasing delay in treatment (37). As an example, Figure 1 shows the estimated survival curve for cardiac arrests as a function of time. The curve demonstrates a steep decline in survival as response time increases, highlighting the critical importance of early treatment initiation.

Table 1 summarizes the average ambulance response times and simulated survival rates across the four municipalities. Large differences in baseline response times were observed, ranging from just over 10 min in Bergen to nearly 33 min in the island municipality of Lurøy. Correspondingly, baseline survival rates varied widely, from 47.7% in Bergen to only 9.2% in Lurøy.

**Table 1:** Average ambulance response times and simulated survival rates (SR) under different early-call scenarios across four Norwegian municipalities.

Municipality	Type	Response time [min]	SR scenario 0	SR scenario 1 (1 min)	SR scenario 2 (5 min)	SR scenario 3 (10 min)
Bergen	Big city	10,2	47,7%	51,2%	68,5%	98,5%
Tokke	Mountain	16,6	29,9%	32,2%	43,0%	61,9%
Lurøy	Island	32,8	9,2%	9,9%	13,3%	19,1%
Sørfold	Rural	22,6	19,4%	20,8%	27,8%	40,0%

SR Scenario 1 = 1 min earlier, SR Scenario 2 = 5 min earlier, SR Scenario 3 = 10 min earlier.

**Figure 2:**
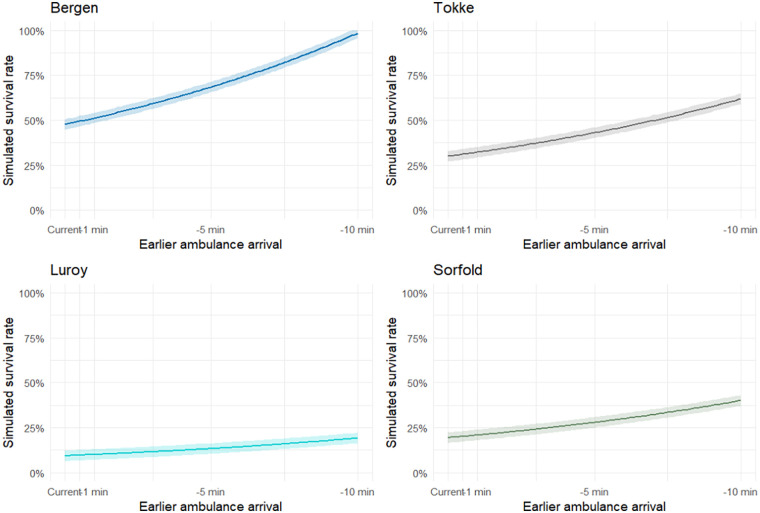
Monte carlo simulations survival curves for the different municipalities, *n* = 10,000 iterations.

The simulations illustrate that even modest reductions in response time yield meaningful improvements in survival (Figure 2). A one-minute earlier call increased survival probability by 3–4 percentage points in Bergen and Tokke, while the effect was smaller but still notable in Sørfold and Lurøy. At five minutes earlier, gains became substantial; Bergen's simulated survival increased from 47.7% to 68.6%, and Tokke from 30.0% to 43.1%. In contrast, the absolute gain in Lurøy remained more modest, increasing from 9.3% to 13.3%. At ten minutes earlier, the relative benefits were most striking. Bergen approached near-universal survival (98.6%), while even municipalities with long baseline delays saw marked improvements. For example, Sørfold increased from 19.4% to 40.1%, and Tokke from 30.0% to 61.9%. Lurøy increased from 9.2% to 19.1%. These findings highlight the strong influence of earlier response on survival, but also the persistent disadvantage of municipalities with long baseline response times.

## Discussion

The present study highlights the critical role of time in determining survival following out-of-hospital cardiac arrest. Using data from four geographically diverse Norwegian municipalities, we observed large disparities in baseline ambulance response times, ranging from approximately 10 min in Bergen to more than 30 min in Lurøy. These differences translated directly into substantial variations in survival probabilities, with baseline estimates of 47.7% and 9.3%, respectively. The simulations further demonstrate that even modest reductions in time to response could yield considerable survival benefits. For instance, a five-minute earlier call increased the simulated survival probability in Sørfold from 19.4% to 27.8%, and increased survival in Tokke by more than 13 percentage points. A 10-minute earlier call nearly doubled the survival probability for the island municipality Lurøy, highlighting the impact in more remote areas. Taken together, these findings emphasize both the importance of early contact with emergency services and the persistent structural disadvantage faced by rural and hard-to-reach municipalities. While reducing ambulance response times through organizational or infrastructural reforms remains challenging in sparsely populated areas (38, 39), strategies that facilitate earlier recognition and action at the patient level may offer a feasible path forward. A key strength of the modelling approach is its transparency; the exponential decay function allows direct comparison across municipalities without introducing assumptions that cannot be validated with currently available data. The framework is deliberately simple so that future studies may expand it by for instance, incorporating weather-related delays, variable terrain, call-handling heterogeneity, or municipality-specific decline rates based on empirical data. Our intention is to provide an open, adaptable modelling template rather than a predictive tool.

In this context, digital health technologies such as wearable devices, home monitoring systems, and AI-driven analytics may play a valuable role (40, 41). Although such tools cannot predict cardiac arrest with certainty, they can identify deviations from an individual's normal physiological state (e.g., arrhythmias, ischemic changes, or abnormal variability) and provide timely feedback (30–32). This may increase patient awareness, encourage individuals at risk to remain prepared, and most importantly, prompt earlier calls for help. As our simulations suggest, even a one- or two-minute advance in contacting emergency services can yield meaningful improvements in survival, while a five- to ten-minute advance can be transformative. Our findings also highlight the importance of tailoring technological solutions to different geographical contexts. In municipalities with already short response times, early calls may push survival probabilities close to theoretical maxima. In contrast, in rural or remote areas with baseline delays of 20–30 min, early recognition may not fully compensate for structural constraints but can still make the difference between survival and death. Wearables and AI could therefore serve as an important complement, but not a replacement, for continued efforts to strengthen emergency preparedness in remote regions.

Finally, this study should be viewed as a proof-of-concept rather than a definitive prediction. The survival estimates are based on simulations using published survival decay rates (5–7), and the results are therefore sensitive to the assumptions embedded in the model. Future work should aim to validate these estimates against real-world data and explore how digital health interventions may be deployed equitably across populations.

### The way forward: a promising strategy to reduce response times

The Norwegian healthcare system has for a long time been under significant pressure, facing an increasing patient load, an aging population, shortages of specialized healthcare professionals, limited capacity, and persistent challenges in recruitment. In this context, technology and innovation represent a necessary part of the solution. The care provided in the very first minutes of an acute illness or injury is often decisive for patient prognosis and survival.

Emerging AI-based technologies have the potential to reduce the time from incident to treatment in emergency medicine. For instance, the EU-funded AI4EMS-project which all the way back in 2020 showed that technology based on speech-recognition and machine learning utilized by Emergency Medical Communication Centers (AMK) could reduce the amount of undetected cardiac arrests by 43% and recognise the most relevant signs for cardiac arrests 25 percent faster than the human call-taker (42). The 2019 Apple Heart Study also demonstrated that consumer smartwatches can reliably detect abnormal heart rhythms (43). However, this study primarily focused on the more common and less time-critical arrhythmia atrial fibrillation (AF). There are now numerous studies and case reports of the Apple Watch detecting AF and more serious arrhythmias such as ventricular tachycardia (44, 45). These reports suggest that AI-enabled wearable devices can prompt users to seek timely medical evaluation. Newer research have also shown that photoplethysmogram (PPG) sensors, the type of sensors most commonly used in consumer wearable devices, can reliably distinguish between pulseless states assosciated with cardiac arrest and non-physiologic states, such as when the wearable is removed from the users body (46). Our findings complement this growing body of work by quantifying the potential survival gains that earlier recognition (regardless of the specific technology used) could provide across municipalities with very different baseline response times. By modeling how even small advances in patient-initiated contact translate into improved survival probabilities, our study offers a generalizable framework that can inform future evaluations of emerging detection systems. Rather than advocating for any single device, we provide an evidence-based context for assessing how innovations in early recognition may differentially benefit urban and rural regions.

In addition to the AI4EMS and wearable technologies described above, a growing range of approaches is being investigated to support earlier recognition of cardiac arrest and other time-critical medical emergencies. Sensor-based systems installed in homes or public spaces can continuously monitor physiological or environmental signals and alert emergency services when abnormal events occur (32, 33, 41). AI-assisted camera surveillance in public areas has shown potential for detecting emergencies such as sudden collapses, enabling rapid mobilization of responders. Smartphone-based alerting systems can connect bystanders or trained volunteer responders to nearby incidents, shortening the time from event onset to initiation of care. Wearable devices, including smartwatches and chest straps, are increasingly capable of real-time physiological monitoring, detecting abnormal heart rhythms, changes in oxygen saturation, or other vital signs indicative of acute events. Each of these technologies varies in terms of feasibility, user adoption, regulatory requirements, and evidence base, and few have been rigorously evaluated for impact on survival after out-of-hospital cardiac arrest. Together, however, they illustrate a rapidly evolving landscape in which early detection and timely intervention may increasingly be achievable. Our study complements this broader technological context by quantifying how modest reductions in the time to patient-initiated emergency contact (regardless of the technology used) can translate into meaningful improvements in survival across municipalities with different baseline response times.

In Norway, a pilot project is currently testing the Aidency 113KIT, a remotely accessible medical emergency kit developed through collaboration between AMK centers, emergency clinicians, and private partners. The kit allows AMK operators to remotely unlock the unit and guide bystanders through selected interventions when clinically appropriate. The ongoing pilot began in late 2024 and includes 35 units located in Møre og Romsdal and Vestfold og Telemark. In 2025, AMK centers activated the kit eight times (47). Early reports document use in cases such as anaphylaxis, hypoglycemia, respiratory distress, and chest pain. These observations are descriptive; systematic evaluation of clinical outcomes has not yet been conducted. The kit contains medications including adrenaline, naloxone, glucagon, acetylsalicylic acid, glyceryl trinitrate, and inhalation medicine, as well as basic trauma equipment. Decisions about activation and use are made solely by AMK personnel, who follow established procedures and receive specific training related to the system. The device was not developed for early detection of cardiac arrest, and its role in such cases remains to be clarified. Norway already benefits from very high rates of bystander-initiated cardiopulmonary resuscitation (CPR), with national data showing initiation in 78% of cases in 2024 (48), compared with an average of 58% across 28 European countries in the EuReCa TWO study (49). Because bystander CPR is strongly associated with improved survival (50, 51), initiatives involving lay responders must be interpreted within this broader context. The pilot aims to assess the feasibility and operational integration of the system in selected emergency situations, particularly in areas with longer prehospital response times. Further research is required to determine safety, effectiveness, and potential impact.

The integration of AI-based technologies and pilot systems such as the 113KIT provides opportunities to explore how early recognition and guided interventions might affect prehospital care; however, the feasibility, safety, and effectiveness of these approaches remain to be rigorously evaluated in future studies.

## Limitations

There are some limitations that should be acknowledged. First, our results are based on simulated survival functions rather than individual-level patient data. While grounded in published estimates of survival decline per minute, the absolute values are sensitive to model assumptions, including the choice of baseline survival probabilities and decay rates. Second, the simulations do not account for variability in bystander CPR, defibrillator availability, or differences in prehospital care quality, all of which influence survival. Third, although digital health technologies hold promise, their adoption may be uneven across populations due to cost, accessibility, or technological literacy, potentially exacerbating existing health disparities. Finally, our analysis focused on four municipalities, which illustrate geographic diversity but cannot capture the full heterogeneity of Norway's healthcare landscape.

## Conclusion

This study demonstrates that even small reductions in time to emergency response can substantially improve survival outcomes following out-of-hospital cardiac arrest. By comparing municipalities with markedly different ambulance response times, we illustrate both the scale of geographical disparities and the potential of earlier patient-initiated contact to mitigate them. While wearables and AI-based monitoring cannot eliminate structural barriers in rural healthcare, they may serve as a valuable complement by promoting earlier recognition of abnormal physiological patterns, thus encouraging timely calls for help. Pilot systems such as the Aidency 113KIT are currently being evaluated for feasibility and integration in prehospital care; their potential role in supporting early interventions remains to be systematically assessed.

## Data Availability

The datasets presented in this study can be found in online repositories. The names of the repository/repositories and accession number(s) can be found below: https://github.com/MariusJohans/s_15.
